# Future perspectives of uveal melanoma blood based biomarkers

**DOI:** 10.1038/s41416-022-01723-8

**Published:** 2022-02-21

**Authors:** Aaron B. Beasley, Fred K. Chen, Timothy W. Isaacs, Elin S. Gray

**Affiliations:** 1 School of Medical and Health Sciences, Edith Cowan University, Joondalup, WA Australia; 2 Centre for Precision Health, Edith Cowan University, Joondalup, WA Australia; 3 Centre for Ophthalmology and Visual Sciences (incorporating Lions Eye Institute), The University of Western Australia, Nedlands, WA Australia; 4 Department of Ophthalmology, Royal Perth Hospital, Perth, WA Australia; 5 Department of Ophthalmology, Perth Children’s Hospital, Perth, WA Australia; 6 Perth Retina, West Leederville, WA Australia

**Keywords:** Eye cancer, Biomarkers

## Abstract

Uveal melanoma (UM) is the most common primary intraocular malignancy affecting adults. Despite successful local treatment of the primary tumour, metastatic disease develops in up to 50% of patients. Metastatic UM carries a particularly poor prognosis, with no effective therapeutic option available to date. Genetic studies of UM have demonstrated that cytogenetic features, including gene expression, somatic copy number alterations and specific gene mutations can allow more accurate assessment of metastatic risk. Pre-emptive therapies to avert metastasis are being tested in clinical trials in patients with high-risk UM. However, current prognostic methods require an intraocular tumour biopsy, which is a highly invasive procedure carrying a risk of vision-threatening complications and is limited by sampling variability. Recently, a new diagnostic concept known as “liquid biopsy” has emerged, heralding a substantial potential for minimally invasive genetic characterisation of tumours. Here, we examine the current evidence supporting the potential of blood circulating tumour cells (CTCs), circulating tumour DNA (ctDNA), microRNA (miRNA) and exosomes as biomarkers for UM. In particular, we discuss the potential of these biomarkers to aid clinical decision making throughout the management of UM patients.

## Introduction and Background

**Fig. 1:**
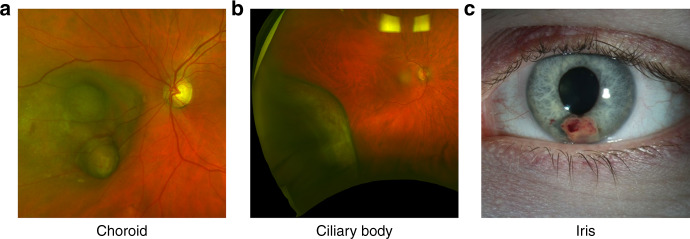
Uveal Melanoma Locations. Macroscopic images of uveal melanoma in the choroid (**a**), ciliary body **(b**), and iris (**c**).

Uveal melanoma (UM) is the most common, and deadliest, form of primary intraocular malignancy affecting adults. Uveal melanomas can arise in any part of the uveal tract, including the iris, ciliary body, and choroid (Fig. 1), but most UM develops in the choroid (~90%) [1]. Initial detection of UM commonly occurs through regular visits to optometrists or ophthalmologists, based on clinical features and irrespective of the presence of typical symptoms such as increased floaters, blurred vision, flashes, or loss of peripheral vision, during routine clinical examinations [2]. Formal diagnosis of UM, however, occurs through clinical examination by an ophthalmologist with experience in ocular tumours (ocular oncologist). Features such as subretinal fluid, orange pigment and documented growth, combined with imaging techniques such as fluorescein angiography and ocular echography are needed to accurately diagnose patients [3].

Early diagnosis is crucial in the treatment and outcome of UM. For example, patients with delayed diagnosis due to misdiagnosis as a naevus or macular degeneration, or failure to detect the tumour during ophthalmoscopy, are more likely to be treated with enucleation rather than more conservative options, such as local resection or plaque radiotherapy [4]. Furthermore, allowing the tumour to grow, even by as little as one millimetre, has been shown to increase the risk of developing metastases by 6–51%, based on its current size [5]. Thus, early detection and treatment interventions are critical for optimal management and improved outcome of this disease.

Here, we summarise the current literature on the clinical management of UM based on histological and genetic features. We discuss the current limitations and highlight how blood-based liquid biopsies could be used in the diagnosis, prognostication, and earlier treatment of UM.

## Current treatment paradigm

Management of UM varies depending on tumour location and size, visual acuity on presentation, local medical expertise and infrastructure available for treatment modalities like proton beam irradiation, and, ultimately, on patient wishes [6]. Iris melanoma is usually treated with radiation, preferably proton beam irradiation, followed by brachytherapy. Although less common, surgical resection is also used [1]. In contrast, treatment for ciliary body and choroidal melanoma varies significantly depending on several ocular and patient factors. Most patients with posterior UM are currently treated with plaque radiation therapy, stereotactic radiosurgery or enucleation. Other available treatment options include proton beam radiotherapy, transpupillary thermotherapy, laser photocoagulation, and rarely, local surgical resection [7, 8]. Overall, the tendency is to treat large, rapidly growing lesions more urgently and aggressively. However, based on evidence suggesting that small primary UM tumours are capable of rapid growth and metastatic spread [5], there is a trend away from observation of small tumours, favouring earlier intervention [9].

Local treatment of the primary UM has improved dramatically in the last 30 years, with increased use of conservative treatments and therapies aimed at preserving the eye [8]. However, survival rates of patients who develop metastatic disease remains dismal, with the 5 year survival rate, from 1973 to 2008, remaining under 10% [10]. This is similar to a recent update reported by Aronow et al. [11] where the 5-year relative survival rate was 80.9% from 1973 to 2013; and our recent report showing no significant change on the 5-year survival rate from 1982 to 2011 in Australia with an average of 81% [12]. This underscores that improvements in the management and control of that the localised disease have had little to no impact on the development of incurable metastases.

Following a diagnosis of UM, patients are screened for metastatic disease, usually with imaging of the liver by ultrasound, magnetic resonance imaging (MRI) and other liver function tests. Positron emission tomography-computed tomography (PET-CT) is also used in some practices [13]. At the time of diagnosis, less than 4% of UM patients have radiologically detectable metastatic disease [14]. However, approximately 50% of patients develop metastatic disease after 2.4–4.4 years [15–18] following diagnosis of the primary tumour. Haematogenous dissemination of UM cells commonly leads metastases towards the liver (93%), followed by lung (24%) and bones (16%) [19] and only rarely to the brain, skin, or other body sites. Metastatic UM is fatal in most cases, with death occurring in 80% of patients within 1 year, and 92% within 2 years of diagnosis of metastases [20]. Therefore, following local treatment of the primary tumour, metastatic screening is generally recommended but is performed at the discretion of the treating ophthalmologist or oncologist, taking into consideration the results of prognostic testing performed and the wishes of the patient.

Demographic, clinical, and histological features of the primary UM have been found to correlate with patient outcome after adjustment for baseline death rates in the general populations [16]. Poor prognostic features include older age at presentation, being male, larger tumour basal diameter and thickness, ciliary body location, diffuse tumour configuration, association with ocular/oculodermal melanocytosis, extraocular tumour extension, and more advanced tumour staging using the American Joint Committee on Cancer classification (AJCC) [21]. In addition, certain histopathological features of the tumour also carry prognostic value, including evidence of epithelioid cell types, high mitotic activity, increased human leucocyte antigen (HLA) expression, tumour infiltration by proangiogenic M2-macrophages (TAMs) and lymphocytes (TILs), higher expression of insulin-like growth factor-1 receptor (IGF1R), microvascular loops and networks, and extracellular matrix patterns [22]. While classically clinical and histopathological features were used to predict patient prognosis, genetics has more recently come to the forefront of UM prognostication.

## Genetic prognostic biomarkers: chromosomal abnormalities, gene expression profiling and driver mutations

Accurate characterisation of the metastatic risk of individual patients is now possible through genetic profiling of UM tumours, which allows identification of changes relevant for prognosis such as somatic copy number alterations (SCNAs), gene expression profiling, or specific gene mutations [23].

### Gene expression profiles

Studies have revealed that mRNA-based gene expression profiling can differentiate two distinct molecular forms of UM cells. Class 1 UMs have a low risk of metastasis, with gene expression similar to normal uveal melanocytes, whilst class 2 UMs harbour high-risk of metastases, with a more primitive stem-like gene expression profile [24]. As a result, a test utilising 15 differentially expressed mRNA, including 12 class discriminating genes, was developed and is commonly used clinically in North America under the name DecisionDx-UM [25]. While the genes selected for the test are associated with loss of chromosome 3 and gain of chromosome 8q, the overall gene expression differences observed between class 1 and 2 are only partially explained by chromosomal alterations [26]. The gene expression profiling test, DecisionDx-UM, has been prospectively validated by two independent multicentre studies, stratifying patients into low-risk class 1 or high-risk class 2 groups. Onken et al. showed from 446 cases analysed, metastases were detected in 3 class 1 cases (1.1%) and 44 class 2 cases (25.9%), over a median of 17.4 months of follow up [27]. Plasseraud et al., showed that class 1 patients had a 100% 3-year metastasis-free survival, whereas class 2 patients had a 63% 3-year metastasis-free survival, with a median time to metastases of 1.4 years [25]. More recently, it was shown that the expression of the preferentially expressed antigen in melanoma (*PRAME*) gene can aid the identification of a proportion of class 1 tumours that give rise to metastatic disease (class 1b) [28]. PRAME is a protein that functions as an inhibitor of retinoic acid signalling, which prevents cell proliferation, differentiation, and apoptosis [29]. Inclusion of *PRAME* gene expression further improved the prognostic predictive power of the DecisionDx-UM test [28]. Thus it should be noted that the study by Plasseraud et al. did not incorporate *PRAME* expression into their classification, with two class 1 patients developing metastases at 3.24 and 4.37 years following diagnosis [25].

### Somatic alterations: copy number variations and somatic mutations

As early as 1990, chromosomal abnormalities were noted in UM cells [30], with loss of one copy of chromosome 3 (monosomy 3) found in approximately 50% of tumours [31]. Monosomy 3 has been reported in 67-84% of metastatic UM [32, 33] and approximately 21% in non-metastasising UM [32]. Furthermore, gain of chromosome 8 affects approximately 40% of UM, and commonly co-occurs with loss of chromosome 3 and metastasis [34], which may or may not co-occur with the loss of 8p, loss of 6q and loss of 1p [35]. In particular, the combination of monosomy 3 and gain of 8q has been shown to confer an extremely poor prognosis [23, 36]. Conversely, other chromosomal alterations are associated with an improved prognosis, including gain of chromosome 6p [37], which is virtually mutually exclusive with loss of chromosome 3 [38]. Based on these SCNAs, a commercially available multiplex ligation probe amplification (MLPA) kit to detect changes to chromosome 1p, 3, 6, and 8 was developed and validated to predict patient prognosis [39]. More recently, a study including 658 primary UM patients, the largest retrospective cohort to date, further demonstrated that SCNA-based classification predicts the risk of melanoma-related metastasis [36], and is more accurate at predicting metastases than AJCC staging [40]. New algorithms such as the Liverpool Uveal Melanoma Prognosticator Online (LUMPO) [41] and Prediction of Risk of Metastasis in Uveal Melanoma (PRiMeUM) [42] combine clinical, histological, and somatic copy number alterations to further refine patient prognostication.

Furthermore, it is known that certain somatic mutations are highly recurrent in UM independently of prognosis, while others co-occur with specific SCNAs and prognostic classes [23]. Somatic mutations to the G Protein Subunit α11 (*GNA11*) and G Protein Subunit αq (*GNAQ*) in codons Q209 and R183 have been known to occur in >90% of UMs [23, 43]. In addition, mutations to the phospholipase C beta 4 (*PLCβ4*) in the codon D630 [44]; and cysteinyl leukotriene receptor 2 (*CYSLTR2*) in the codon L129 [45] were found in GNAQ/GNA11 wild-type UMs. The above mutations are largely mutually exclusive and found within the same cellular pathway [45]. Therefore, they are thought to constitute the means for UM cells to achieve constitutive activation of the mitogen activated protein kinase pathway (MAPK) and the phosphatidylinositol-4,5-bisphosphate 3-kinase (PI3K)/protein kinase B (AKT) pathway.

Although mutations to *GNA11*, *GNAQ*, *PLCβ4*, and *CYSLTR2* do not correlate with prognosis, somatic mutations to the de-ubiquitinating enzyme BRCA1 associated protein 1 (*BAP1*), splicing factor 3B subunit 1 (*SF3B1*), and eukaryotic translation initiation factor 1A X-linked (*EIF1AX*) are associated with prognosis. Inactivating somatic mutations to *BAP1* are found in 44–66% primary UM [23, 43] and in around 84% of UM metastases, and are associated with poor prognosis [23, 46]. Inactivation of *BAP1* has been shown to induce a metastatic phenotype in vitro and is associated with low oxidative phosphorylation and reliance on fatty acid oxidation [47]. Furthermore, the location of *BAP1* on chromosome 3p may explain the association between loss of chromosome 3 and inactivation of *BAP1* on the remaining chromosome 3. In contrast, mutations to *SF3B1* have been shown to correlate with intermediate prognosis and disomy of chromosome 3. Similarly, mutations to *EIF1AX* are also associated with disomy of chromosome 3 and shown to be correlated with a good prognosis [48].

**Fig. 2:**
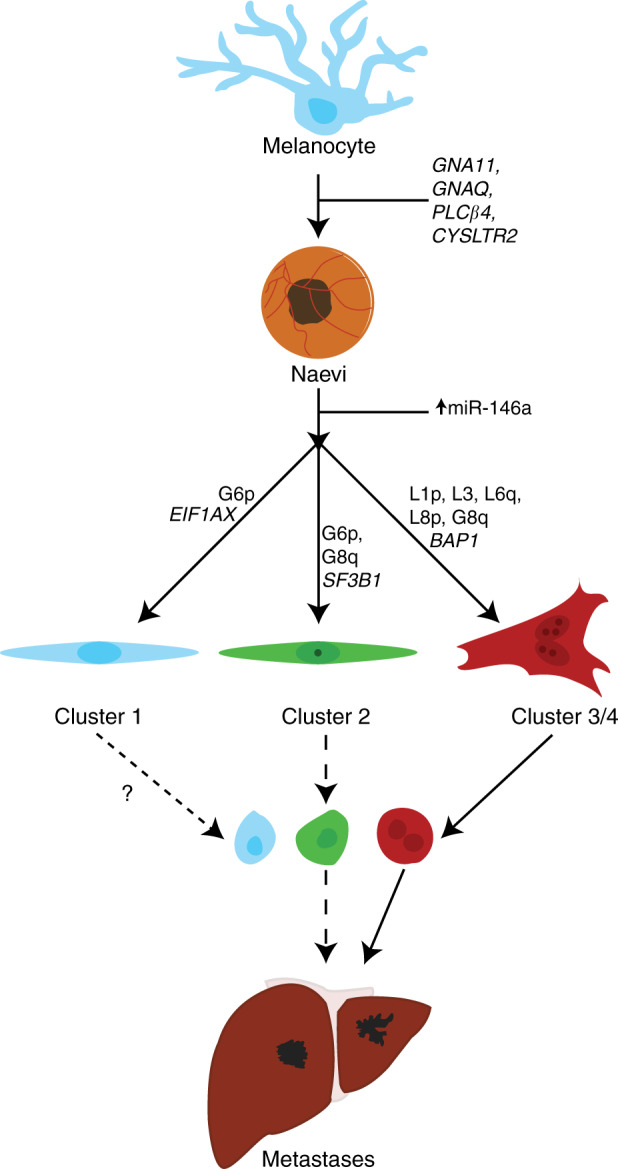
Genetic pathways of uveal melanoma progression. Non-prognostic mutations to *GNA11*, *GNAQ*, *PLCβ4*, and *CYSLTR2* may drive the transformation from normal uveal melanocyte to early UM. Further genetic abnormalities dichotomise uveal melanoma into several prognostic groups. Patients with mutations to *EIF1AX* generally have the best prognosis and have no SCNA or G6p. Patients with mutations to *SF3B1* have an intermediate prognosis and have G6p and/or abnormalities to chromosome 8. Lastly, patients with mutations to *BAP1* have loss of chromosome 3 and gain of 8q, with larger gains in 8q equating to a shorter time to metastases. Each class of tumour may release CTCs at different rates and quantities, and may cause the formation of metastases.

A comprehensive integrative analysis by The Cancer Genome Atlas Program revealed a strong association of somatic alterations and gene expression profiles, which define four molecularly distinct clinically relevant subtypes of UM. These SCNA clusters were further validated by Vichitvejpaisal et al. in 2019 in 658 eyes at the Wills Eye Hospital. Thi*s* study referred to these as clusters A–D rather than 1–4 as used in the TCGA study [36]. Cluster 1 tumours are characterised by *EIF1AX* mutations, gain of chromosome 6p or neutral SCNA profiles; and are associated with good prognosis, with only 4% of patients developing metastases after 5 years from diagnosis [36]. Cluster 2 tumours carry *SF3B1* mutations and gain of chromosome 6p, loss of chromosome 6q and gain of chromosome 8q, and are associated with a 5-year metastatic risk of 20%. Lastly, cluster 3 and 4 tumours are associated with poor prognosis and carry *BAP1* mutations and loss of chromosome 1p, loss of chromosome 3, loss of chromosome 6q, loss of chromosome 8p and gain of chromosome 8q. (Fig. 2) [23, 36]. The risk of developing metastases by 5 years, increased from 33% to 63% depending on the level of 8q gains, and therefore, further subdivided as cluster 3 and cluster 4, respectively [36].

As a result of the overwhelming evidence, the new guidelines set out by the National Comprehensive Cancer Network (NCCN) incorporated the aforementioned clinical, histopathological and genetic features to provide clinicians with an up to date treatment and care guidelines [6]. Patient prognostication by gene expression profiling and/or SCNA, is critical to inform systemic imaging frequency based on the highest risk factor present. Surveillance imaging frequency varies from yearly to three-monthly radiological scans, for low and high-risk groups, respectively. As no systemic therapies that can improve overall survival are currently available, once metastases are detected, the primary directive is to enrol patients into clinical trials or liver-directed therapies.

## Tumour sampling and implications of prognostic testing

Fine-needle aspiration biopsy (FNAB), usually performed at the time of plaque brachytherapy, is the most frequently used technique for biopsy of UM for molecular analysis. However, incisional biopsy, with or without vitrectomy surgery, is also performed routinely in some centres. The biopsy technique chosen depends upon the size, configuration, and most importantly the location of the tumour. For iris tumours, entry into the anterior chamber can be accomplished using a 26- to 30-gauge needle under direct visualisation with an operating microscope [49]. The needle is inserted bevel up and is swept gently over the surface of the tumour, while approximately 0.5 mL of aqueous fluid containing the specimen cells is aspirated. Posterior-segment tumours can be biopsied via a transscleral or a transvitreal approach. The transscleral approach, in which the biopsy needle is passed through the sclera and into the tumour, is the most commonly employed, and is suitable for biopsy of UM located within the ciliary body or the anterior choroid [50]. Alternatively, an incisional approach can be used, in which a partial thickness scleral flap is fashioned, and the choroid exposed by a full-thickness scleral incision, allowing biopsy forceps to capture a much larger specimen than can be obtained using FNAB techniques. Tumours located posterior to the equator can be approached via a transretinal technique through the vitreous cavity, either by FNAB via the pars plana, or via vitrectomy surgery.

Excisional biopsy, using the technique of lamellar sclerouvectomy, is performed infrequently, since most UM are unsuitable due to size, configuration or location. This technique can achieve complete local tumour control and excellent visual outcomes. However, the surgery is technically challenging, requires prolonged hypotensive anaesthesia, and carries a significant risk of complications including vitreous haemorrhage [51], rhegmatogenous retinal detachment, and local recurrence (25–35%, 10 years) [52].

Whilst FNAB can provide useful diagnostic and prognostic information, all biopsy techniques carry the risk of complications, including haemorrhage and infection, which may be sight-threatening. Furthermore, insufficient material for diagnostic purposes has been reported in ~8–20% of FNAB cases [50, 53]. A large prospective interventional case series (*n* = 150) indicated that transvitreal or transcorneal routes have higher rates of failure compared to the transscleral route, presumably due to lower tissue yield. Moreover, negative results were significantly correlated with small tumour sizes (basal diameter <5.0 mm; apical height <2.5 mm) [50]. This likely reflects the clinical difficulty of obtaining sufficient biopsy material from smaller tumours. Current evidence suggests that early treatment of UM leads to improved patient survival [5]. However, prognostic testing may be more difficult to perform in these smaller tumours due to lower specimen yield. Additional factors that may reduce accurate prognostication include tumour heterogeneity and sampling errors [54].

Local seeding of tumour cells along the biopsy track has been raised as a possible risk of UM biopsy, and appears to be rare, however local recurrence of melanoma following FNAB has been reported [55]. In a study of 170 FNABs, there were no reported cases of local treatment failure, endophthalmitis or orbital dissemination. Metastatic disease developed in 14 patients and retinal detachment occurred in 3 cases, but none of these were directly associated with FNAB [56].

There is a demonstrable need for alternative methods of prognostic testing for patients with UM. Recently, we described accurate identification of SCNAs in primary FFPE UM tumour samples using whole genome amplification and low-pass whole genome sequencing [57]. This method could support current clinical practice to help overcome failure rates due to sub-optimal tissue yields from FNAB.

Prognostic testing of UM carries psychological consequences for patients. With rare exceptions, systemic therapy does not reduce mortality or prolong survival in patients with metastatic UM. Nevertheless, studies have shown that the majority of patients wish to know prognostic information about risk of metastasis, even though no accepted and effective treatments exist for disseminated disease. The most important reason for UM prognostication is to provide patients with information that would be helpful in making life-changing decisions [58]. A recent study evaluating the psychological needs of patients discovered that of 261 UM patients, 79.5% needed psychological intervention [59].

## Blood biomarkers

**Fig. 3:**
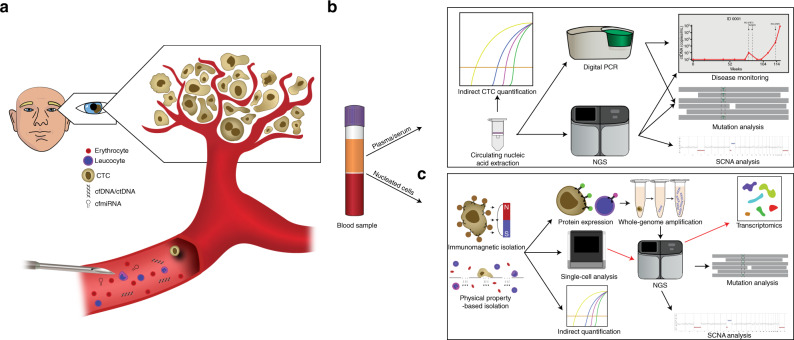
Liquid biopsy. **a** CTCs, ctDNA, and cmiRNA are minor components of the blood, mixed with the normal erythrocytes, leucocytes, and cell-free nucleic acids. A minimally invasive venous blood collection has the potential to provide genetic information of all tumours within the body. **b** Plasma derived circulating nucleic acids (DNA, RNA, miRNA) are generally extracted using commercially available kits. Following extraction, ctDNA or cmiRNA can be detected by real time-qPCR, digital PCR or NGS, to detect the presence of disease, to detect mutations or SCNAs for diagnostic, prognostic, or genetic changes in response to therapy. **c** Circulating tumour cells are isolated from the blood by antibody directed methods such as immunomagnetic beads, or through their physical properties such as size and deformability. Once isolated, CTCs can be indirectly quantified using RT-PCR, or directly quantified using immunocytochemistry. Fluorescence in-situ hybridisation (FISH) can be used to detect SCNAs or changes to mRNA or miRNA expression. Single cell sequencing of CTCs can provide mutational and/or chromosomal copy number information.

A growing number of publications, including our own, have documented that tumour derived material can be detected in the circulating blood, serving as “liquid biopsies” (Fig. 3a). Liquid biopsies provide a minimally invasive alternative for diagnosis, prognosis, and monitoring of cancer. In particular, circulating biomarkers such as circulating tumour cells (CTCs), circulating tumour DNA (ctDNA), and Circulating Micro RNA (cmiRNA) have been explored for the management of UM through the application of new technologies (Fig. 3b, c) in combination with clinical studies.

### Circulating tumour cell enumeration

CTCs are tumour cells that have disseminated into the vascular or lymphatic system of cancer patients, believed to facilitate the formation of metastases. Histopathological analysis of 643 eyes enucleated from patients with UM demonstrated that intravascular ingrowth of tumours occurred frequently with other well-studied histopathological features of poor prognosis (epithelioid cell type, intrascleral growth and large tumour size) [60]. It is generally accepted that the eye has no well-developed lymphatic drainage [61]. This may suggest that a haematogenous route of dissemination through the release of CTCs might be common in UM and may be a critical step in the process of metastasis.

Studies across multiple malignancies have demonstrated that CTCs may be used to predict prognosis, response to therapy and disease recurrence [62]. For example, in castrate-resistant prostate cancer patients highlighted the clinical utility of AR-V7 variant identification on CTCs to inform and improve treatment outcomes, relative to decisions based on current standard of care [63]. A recent meta‐analysis of 21 studies showed that CTC enumeration as a prognostic marker in non-metastatic breast cancer patients [64]. Moreover, detection of CTCs before neoadjuvant chemotherapy was significantly associated with reduced overall, distant disease-free and locoregional relapse-free survival [64]. In addition, a pooled analysis of 18 cohorts supported the use of CTC enumeration for staging of metastatic breast cancer [65].

A recent large study in cutaneous melanoma revealed that detection of ≥1 CTC in stage III patients was a significant predictor of decreased 6-month and 54-month relapse-free survival [66]. Such a test could be used to identify patients who may benefit from adjuvant therapies. Moreover, we have demonstrated the heterogeneous nature of cutaneous melanoma CTCs [67], that CTC subpopulations respond dynamically to therapy [68] and that the presence of specific CTC phenotypes is predictive of response and progression-free survival [68]. All the above underscores the potential of CTCs as a cancer biomarker.

Detection of CTCs in UM was originally accomplished using cytochemistry and standard microscopy techniques [69, 70]. Initially, the wide-spread use of reverse transcriptase-polymerase chain reaction (RT-PCR) was used to detect UM specific mRNA transcripts. These studies targeted *tyrosinase*, melanoma antigen recognised by T-cells 1 (*MART1*) and glycoprotein 100 (*gp100*), and more recently, markers of epithelial to mesenchymal transition (Supplementary Table 1) [71–81]. In general, RT-PCR based gene expression has been associated with worse outcomes in primary UM [71, 73–75, 77, 78], but the number of patients with detectable CTCs has been low with a couple of studies not detecting CTCs in primary UM [72, 81]. Lastly, there are limited studies assessing metastatic UM CTCs, but generally CTCs are readily detectable [71, 74, 79], and their presence has been shown to be associated with shorter PFS and OS [79].

Although RT-PCR is a relatively straightforward technique to indirectly infer the existence of CTCs, it does not provide conclusive evidence of the presence of CTCs. Furthermore, RT-PCRs do not provide phenotypic or genotypic information of the CTCs, nor can it differentiate between CTCs and circulating RNA. As an alternative, direct capture and enumeration of CTCs have been evaluated as prognostic biomarkers in UM patients. Immunomagnetic isolation, using covalently conjugated antibodies directed against common UM surface antigens bound to ferric fluids or beads, is the most common method utilised today for CTC enrichment. Reports on the direct quantification and characterisation of CTCs by immunomagnetic capture and size-based isolation are listed in Table 1.

**Table 1:** Direct CTC detection studies in UM.

Study	Study Type	Enrichment method	Capture/ Detection Marker	Blood Tube	Blood Vol (mL)	TTP (hrs)	Detection Rate	# CTCs	Summary of outcomes
Ulmer [100]	P	IMC	MCSP/MSCP	H	50	I	NA	NA	aCGH confirmed SCNAs in UM CTCs. Cutaneous and uveal CTC numbers were pooled together.
Ulmer [84]	P	IMC	MCSP/MCSP	H	50	I^b^	10/52 pUM	2.5 (1–5)	CTC detection was associated with ciliary body involvement, advanced tumour stage, and anterior anatomical location
Eide [82]	P	IMC	MCSP, CD276/ LM	H	40	NS	4/249 pUM	NS	CTCs were detected in bone marrow. 98/328 positive with a median of 56 and a range of 1–500 cells detected.
Pinzani [78]	P	ISET	NA/H&E	ISET: EDTA	ISET: 10	≤4	ISET: 5/16 pUM	2 (0.75–5.8)	ISET CTCs correlated with tyrosinase levels, and where tyrosinase was negative, ISET CTCs were negative.
Suesskind [83]	P	IMC	MCSP/MCSP	H	50	I^b^	BL: 13/94 pUM FU: 9/94 pUM	BL: 1 (1–8) FU: 7.5(1–26)	No significant difference in CTC numbers between pre- and -post treatment of the primary tumour. No association between CTCs and prognostic features.
Tura [86]	P	IMC	CD63, gp100/ MCSP, CD63	Li-H	50	≤3	29/31 pUM	3.5/10 mL (0–10.2)	CTCs were also identified after short-term culture.
Mazzini [95]	P	ISET	NA/H&E	EDTA	10	≤3	17/31 pUM	8 (2–50)	CTC clusters are detectable through ISET. > 10 CTCs was indicative of larger LBD, apical height, and disease-free survival.
Bidard [89]	P	IMC-CS	MCAM/MCSP	CS	7.5	≤72	12/40 mUM	(1–20)	CTC count was strongly associated with the presence of miliary hepatic metastases, metastases volume, ctDNA level, PFS, and OS.
Bande [88]	P	IMC-CS	MCAM/MCSP	CS	7.5	≤72	4/8 pUM	0.5 (1–3)	No correlation between CTC positivity and LBD & apical height.
Terai [90]	P	IMC-CS	MCAM/MCSP	CS	7.5	≤72	AB: 17/17 pUM VB: 9/17 pUM	AB: 5 (1–168) VB: 1 (2–17)	Arterial blood was far superior to venous blood for detection of CTCs. Patients with both hepatic and extra-hepatic metastases showed significantly more CTCs in arterial blood compared to liver alone.
Tura [93]	NS	IMC	CD63, gp100/ CD63, MCSP	Li-H^a^	50^a^	≤3^a^	40/44 pUM	2.4/10 mL (0–10.2)	58% of 44 cases were positive for monosomy 3. 11 patients were tested for monosomy 3 in CTCs compared to tissue using immune-FISH. 10/11 CTC monosomy matched the tumour. Monosomy 3 in CTCs correlated with advanced tumour stage and was detected in all 4 patients who developed metastases in the study period.
Beasley [85]	P	IMC	MCSP/MART1, gp100, S100β	EDTA	8	≤24 (held at 4 °C)	18/26 pUM; 1/1 mUM	pUM: 2/8 mL (1–37)	One mUM case was used to show SCNAs in UM CTCs matching the primary tumour. In pUM, CTCs counts did not correlate with tumour size or risk indicators.
Anand [87]	P	IMC-CS	MCAM/MCSP	CS	7.5	≤72	BL: 6/20 pUM 8/19 mUM FU: 8/19 pUM 13/19 mUM	BL: pUM: Mean 1.73 (1–3) mUM: Mean 9 (1–38)	Landmark OS rate at 24 months was significantly lower in CTC positive pUM.
Maaßen [92]	R	IMC	CD63, gp100/ CD63, MCSP	Li-H^a^	50^a^	≤3^a^	19/20 pUM	9.3/50 mL (0–51)	Systemic metastases were associated with the presence of monosomy-3, measured by immune-FISH, in the primary tumour and CTCs as well as a higher GLUT1 ratio.
Tura [94]	NS	IMC	CD63, gp100/ CD63, MCSP	Li-H^a^	50^a^	≤3^a^	30/33 pUM	8.2/50 mL (0–51)	CTCs with monosomy 3 had significantly less Adiponectin in their pUM compared to CTC-ve or CTCs with a lower percentage of monosomy 3.
Beasley [91]	P	IMC	ABCB5, gp100, MCAM, MCSP/ MART1, gp100, S100β	EDTA or TransFix	8	EDTA ≤1 Transfix 1–72	37/43 pUM	3/8 mL (1–89)	≥3 CTCs was associated with shorter PFS and OS.

*TTP* time to process, *NA* not applicable, *P* prospective, *R* retrospective, *NS* not specified, *I* immediately, *IMC* immunomagnetic capture, *IMC-CS* immunomagnetic capture-cell search, *RT-PCR* reverse transcriptase-PCR, *LM* light microscopy, *pUM* primary uveal melanoma, *mUM* metastatic uveal melanoma, *H* heparinised, *EDTA* ethylenediaminetetraacetic acid, *PG* PAXgene, *CS* CellSave, *ISET* isolation by size of tumour cells, *AB* arterial blood, *VB* venous blood. Unless otherwise specified, venous blood was used, *H&;E* ; haematoxylin &; eosin, *BL* baseline, *FU* follow-up, *PFS* progression-free survival, *OS* overall survival, *CTC* counts are displayed as “median (range)” unless otherwise specified.

^a^Assuming based on Tura et al. [86].

^b^Inferred based on Ulmer et al. [100].

Commonly, the melanoma associated chondroitin sulphate proteoglycan (MCSP, also known as chondroitin sulphate proteoglycan 4—CSPG4, high molecular weight-melanoma associated antigen—HMW-MAA or neural glial antigen 2—NG2) has been used as target for capture of UM CTCs. Using this approach, CTCs were detected from 40 to 50 mL of peripheral blood in 1.6–19% of patients with primary UM [82–84], and their detection in one study correlated with known clinical features of poor prognosis [84]. In a follow up study from Ulmer et al. 2008, Suesskind et al. 2011 used immunomagnetic isolation of with MCSP alone but not find any correlation between the quantity of CTCs and the metastatic propensity of the tumour over a median follow up time of 16 months [83]. In our recent study, we used MCSP to isolate CTCs in 69% of primary UM patients prior to treatment from only 8 mL of blood. We found that the presence of CTCs was not associated with tumour size or prognostic class [85]. Similar findings have been reported by Tura et al. [86], potentially indicating that the haematogenous dissemination of tumour cells is not a determinant of metastasis in UM. Rather, cellular programs that underpin the molecular classes drive metastases by enhancing metastatic colonisation, primarily to the liver. In addition to MCSP, immunomagnetic isolation using melanoma cell adhesion molecule (MCAM) has been utilised in the isolation of CTCs in UM patients using the CellSearch system [87–90]. In primary cases, capture rate has ranged from 30 to 50% [87, 88] with some evidence that enumeration of CTCs in early stage UM predict increased metastatic risk and increased mortality [87]. The above results suggest that targeting only one surface antigen is not enough to capture CTCs in most UM patients. To overcome this limitation, CD63 and gp100 were used in combination for immunomagnetic enrichment, and CTCs were detected in 94% of 31 UM patients from 50 mL of peripheral blood [86]. Furthermore, we have also utilised a multimarker CTC isolation approach, targeting ABCB5, gp100, MCAM, and MCSP and found that CTCs were detectable in 86% of 43 primary UM patients from 8 mL of blood correlating with shorter PFS and OS [91]. Indeed it appears as though currently the combination of CD63 and gp100 or for the isolation of CTCs in primary UM is the most suitable capture technique, given that all studies from this group have achieved >90% detection rate [86, 92–94] with median cell counts >1.66 per 10 mL of blood. Lastly, in addition to RT-PCR and immunomagnetic isolation, primary UM CTCs have been isolated using filtration with a detection rate of 31–54% patients with localised disease [78, 95]. Using the ISET filtration system, it was shown that more than 10 CTCs per 10 mL of blood were an indicator of poorer prognosis over a 24 month period [95].

Even though most CTC isolation studies have focused on primary disease, some studies also have investigated metastatic UM. Initially, Bidard et al. 2014 described the use of the CellSearch system to detect CTCs in metastatic UM where CTCs were detected in 30% patients and CTC count was strongly associated with the presence of miliary hepatic metastases, the total volume of disease, progression-free survival, and overall survival [89]. CellSearch was also used to compare CTC counts between arterial and venous blood from metastatic UM patients. CTCs were detectable in 100% of arterial blood compared to 53% in paired venous blood, with higher median cell counts and absolute ranges [90]. More recently, Anand et al. 2019 found CTCs in 42% of metastatic patients compared to only 30% at the primary localised stage [87].

Despite the variety of CTC detection methodologies, there is conflicting evidence on whether the enumeration of CTCs is prognostically relevant in metastatic UM [89]. The combined data indicates that CTCs are present in the blood of UM patients with primary disease. Given the opposing results of some studies, further investigation is needed to understand if a subtype of CTCs, isolated by some specific methods, is associated with high-risk UM and, therefore, prognostically relevant. Interestingly, circulating melanocytes or “CTCs” have not shown to be detectable in patients with ocular naevi [88, 95], which may identify cases at risk of malignant transformation. Thus, this may present an opportunity for minimally invasive diagnosis, but given the limited reports further validation is required.

Many different factors may affect the recovery and detection of CTCs (Table 1, Supplementary Table 1) outside of the specific detection system. For example, the use of fixative tubes such as Streck or CellSave have a marked improvement on CTC recovery when analysis is delayed [96], but in general EDTA is suitable if processing occurs quickly after blood draw. In UM, studies that have utilised the CellSearch have opted for the companion CellSave tubes, whereas others have used EDTA or heparin. In vitro analysis of spiked “CTC mimic” in EDTA tubes showed significant reduction of recovered cells after 1 h [97]. However, storage temperature was not noted in this study which may also be a crucial factor as it is in ctDNA. Other important factors include the input sample type, such as from whole blood (ISET/CellSearch), white blood cells after red blood cell lysis, or peripheral blood mononuclear cells, which may all potentially confound isolation and analysis due to different background levels and types of leucocytes and this has been quite varied historically in UM.

### Circulating tumour cells genetic analysis

Beyond enumeration, CTCs can be used as accessible samples to analyse the genetic profile of the tumours in an individual. Previous work has indicated that CTCs can be used to detect tumour-specific mutations and SCNAs. For example, in small-cell lung cancer, SCNAs found in CTCs that could with reasonable certainty predict whether a tumour would respond to chemotherapy [98]. Similarly, in a pan-cancer study incorporating colon, lung, gastric, and prostate cancer, tumour-specific mutations could be sporadically detected in CTCs as well as focal SCNAs in the *MYC* and *PTEN* genes in all CTCs which were only detected in minor fractions of tumour cells within the primary tumour, possibly indicating selection for metastases [99].

An early report showed the analysis of SCNAs from single UM CTCs using array comparative genomic hybridisation (aCGH). In this study, the authors found that the aCGH profiles of the MCSP positive UM CTCs had aberrations indicative of UM, such as monosomy of chromosome 3. However, these cells were not compared to the tumour of origin [100]. Another study utilised fluorescence in-situ hybridisation (FISH) targeting chromosome 3 and found that monosomy 3 could be detected in CTCs in 53% of patients. Somatic copy number alterations to chromosome 3 in CTCs and tumours were found to be highly concordant, with 91% of the matched tumours and CTCs harbouring the same chromosome 3 status [93]. Similarly, we showed that whole genome amplification (WGA) combined with low pass whole genome sequencing (LP-WGS) can be used to profile SCNA on UM CTCs. The CTCs harboured profiles highly concordant with the primary tissue [85]. Our results are supported by evidence that UM genetic mutations and chromosomal aberrations found in the primary tumour are also present in the patient-matched metastases [101], as UM is regarded as genetically stable disease [102]. The results of these studies support a model for detection of SCNAs associated with UM prognosis through the analysis of CTCs. However, this method needs rigorous clinical validation before it can be used as an alternative prognostic test.

### Circulating tumour DNA

Another blood based biomarker, increasingly attracting attention, is circulating tumour DNA (ctDNA) (Fig. 3a–c) [103]. ctDNA forms as a result of apoptosis or necrosis of tumour cells, or actively secreted from tumour cells thus reflecting the evolving genetic alterations of all solid tumours present at any one time within a patient [104]. Clinically, ctDNA analysis can provide critical clinical information. For example, in stage II colon cancer, previous research has shown that ctDNA detection after resection provides evidence that residual disease remains, that this identifies patients at a high-risk of relapse, and could be used to guide the use of adjuvant chemotherapy [105]. Not only can the quantification of plasma ctDNA provide clinicians with treatment guidance, but also the presence of druggable driver mutations in tumours. In non-small cell lung cancer, a common marker of resistance to EGFR tyrosine kinase inhibitors is the *EGFR* T790M mutation. Detection of this mutation in the plasma ctDNA by the FDA approved “*cobas EGFR Mutation Test v2*” can be used to guide treatment selection, allowing for treatment with Osimertinib [106]. More recently, the approval of Guardant360 CDx by the FDA as the first liquid biopsy test that uses next-generation sequencing marks a new era for mutation testing in oncology [107].

For cutaneous melanoma, ctDNA have been shown to correlate with tumour size, accurately represent tumour mutations, detect recurrence [108, 109] and treatment failure prior to radiological imaging [110–112]. In addition, ctDNA detection can be used to identify stage III melanoma patients at high-risk of relapse [113, 114]. In UM, more than 95% of patients harbour mutually exclusive recurrent mutations affecting *GNA11* (Q209, R183), *GNAQ* (Q209, R183), *PLCβ4* (D630), or *CYSLTR2* (L129) [23, 43]. Although these mutations are not prognostic, they allow for the detection of ctDNA in most patients, using a targeted approach. However, there is limited literature on the use of ctDNA detection. Reports on the detection and quantification of ctDNA in UM patients are listed in Table 2.

**Table 2:** ctDNA studies in UM.

Study	System	Blood Tube	Volume (mL)	TTP (hrs)	Centrifugation (xg/mins)	Extraction Kit	Elution Vol (µL)	Detection rate	ctDNA	Summary of outcomes
Madic [122]	bi-PAP	EDTA	5	≤3	820/10 → 16,000/10	Q + 1 µg carrier RNA	NS	20/21 mUM	(1.3–2125)	ctDNA correlated with tumour burden. The only undetectable sample had a tumour volume of 0.43 cm^3^.
Metz [121]	NGS (no UMI)	EDTA	1–5	≤0.5	1500/10	Q	40	9/28 mUM	0.03–38.4% FA	ctDNA detection correlated with bone metastases. ctDNA detection correlated with younger age of patients and larger metastases.
Piperno-Neumann [124]	bi-PAP	EDTA^a^	5^a^	≤3^a^	820/10 → 16,000/10^a^	Q + 1 µg carrier RNA^a^	NS	51/54 mUM	30 (1–11,421)	Abstract. Correlated with mUM tumour burden.
Bidard [89]	bi-PAP	EDTA	5	≤3	820/10 → 16,000/10	Q	36	18/22 mUM	10 (2–9423)	ctDNA level correlated with hepatic miliary metastases, CTC positivity, and tumour volume. ctDNA levels were an independent prognostic factor for PFS and OS.
Weight [118]	Guardant360 CDx	Streck^c^	5–30 ng^c^	NS	NS	NS	NS	0/32 high risk surveillance 1/10 newly developed muM 18/24 muM	Not stated	Abstract. MRI was found to be more sensitive than ctDNA for detection of metastases. Only lesions >2 cm could be detected.
Cabel [123]	bi-PAP	EDTA	5	≤1	820/10 → 16,000/10	Q	36	BL: 2/3 mUM FU: 2/3 mUM	BL: 0.5 (0–607) FU: 3.7 (0–457)	Included NSCLC, UM, and MSI-instable colorectal cancer.
Beasley [85]	ddPCR	EDTA	1–5	≤24 (held at 4 °C)	300/20 → 1600/10	Q	1-3 mL: 30 4-5 mL: 40	8/30 pUM 8/8 mUM	pUM: 0 (0–29) mUM 178 (2–15,160)	ctDNA significantly higher in mUM compared to pUM. ctDNA detection in pUM correlated with tumour size. ctDNA in a one patient case study was detectable prior to detection of metastases by PET-CT.
Rodrigues [126]	bi-PAP	EDTA	5	≤3	820/10 → 16,000/10	Q + 1 µg carrier RNA	NS	1/1 mUM	NS	Case study. ctDNA was able to track the response of a patient with MBD4 mutation to anti-PD1 therapy. ctDNA levels changed with response to therapy.
Soltysova [81]	ddPCR	EDTA	3	≤4	1500/10 → 3000/10	Q	70	pUM 3/32 mUM 6/11	pUM 0 (0–0.13) mUM 0.3 (0–108) copies/µL	mUM had significantly more ctDNA than pUM.
Park [125]	ddPCR or NGS (with UMI)	EDTA	1–4	≤4	800/15 → 1600/10	Q	25	16/17 mUM	157.7 (Range 0–7172)	Baseline ctDNA correlated with LDH and tumour size. Lower median baseline ctDNA levels correlated with the clinical benefit group. ctDNA predicted response to targeted therapy and increasing ctDNA preceded radiological progression with 4–10-week lead time.
Bustamante [115]	ddPCR	PG or SST	2	≤1	2000/20 → 2000/20	Q	25	14/14 pUM 8/16 naevi	pUM 3.75 (0.7–31.4) Naevi 2.3 (1–13.3)	ctDNA levels correlated with malignancy. In naevi, ctDNA levels correlated with clinical risk factors.
Le Guin [116]	NGS (no UMI)	EDTA	1–5^b^	≤0.5	1500/10^b^	Q^b^	40^b^	3/135 pUM 17/21 mUM or recurrence	0.1–10% FA	ctDNA was commonly detected in mUM compared to pUM. The presence of ctDNA was associated with metastases with 80% sensitivity and 96% specificity.
Francis [117]	NGS	NS	NS	NS	NS	NS	NS	1/1 mUM	*EIF1AX* – 1.14% FA *GNAQ –* 1.49% FA	Case study. Utilises MSK-ACCESS. A patient was monitored every 6 months using MRI. The patient had ctDNA assessed 3 months after an MRI and found ctDNA which triggered earlier reimaging revealing metastatic disease.

ctDNA levels at baseline, unless otherwise specified. All studies were prospective. ctDNA levels displayed as “median (range)” in copies/mL unless otherwise specified.

*TTP* time to process, *Q* QIAmp circulating nucleic acid, *EDTA* ethylenediaminetetraacetic acid, *PG* PAXgene, *FA* fractional abundance, *UMI* unique molecular identifier, *NS* not specified, *pUM* primary uveal melanoma, *mUM* metastatic uveal melanoma, *bi-PAP* bidirectional pyrophosphorolysis-activated polymerisation, *NGS* next-generation sequencing, *ddPCR* droplet digital PCR, *PFS* progression-free survival, *OS* overall survival.

^a^Assumed from Madic et al. [122].

^b^Assumed from Metz et al. [121].

^c^Based on Guardant360 CDx guidelines.

In primary UM, a recent study ctDNA was detected in 100% of primary UM, and 50% of ocular naevi, with ctDNA levels correlating with malignancy [115] using droplet digital PCR (ddPCR) to detect mutation in *GNAQ*, *GNA11*, *PLCβ4*, and *CYSLTR2*. In contrast, in our previous study ctDNA was only detectable in 26% of patients with primary UM prior to localised therapy [85] using ddPCR, and did not correlate with clinical or histological markers of disease. However, there was a slight trend toward detection with increased tumour size. Other recent studies confirmed this finding, showing ctDNA detection in only 2% (3/135) [116] using ultra-deep amplicon sequencing, or 9% (3/32) using ddPCR [81] targeting known mutations in primary UM cases. Thus, the detectability of ctDNA in primary UM is still controversial and requires further investigation.

Currently, studies monitoring ctDNA in UM patients after resolution of the primary lesion are conflicting. Some have shown that ctDNA detection preceded the clinical diagnosis of metastasis by 2–10 months [85, 116, 117]. In one of our case studies, ctDNA detected metastases in a patient with low-grade lymphoproliferative disease and pulmonary embolism, which caused interference of UM detection through PET-CT [85]. Conversely, one study utilising Guardant360 CDx showed ctDNA was less sensitive than MRI [118] at detecting metastatic disease. However, extensive prospective studies with standardised radiological comparators are required to validate the clinical utility of ctDNA for monitoring of high-risk UM patients for early evidence of metastases. A key consideration for the use of ctDNA for early detection of disease relapse is the minimum tumour size/burden and ctDNA shedding by higher tumour turnover. We have shown in cutaneous melanoma that ctDNA only becomes consistently detectable at a metabolic tumour burden of >10 total lesion glycolysis units or metabolic tumour volume of >4 mm^3^, both values calculated from ^18^F-labelled fluorodeoxyglucose PET-CT scans [109]. Nevertheless, assay sensitivity may be improved through the optimisation of pre-analytical, analytical and bioinformatic practices [119]. In our experience, another key component of ctDNA detectability is the location of disease. For example, in cutaneous melanoma we found that patients with intracranial metastases harbour no detectable ctDNA [120]. In contrast, we observed that lesions arising in the liver were generally readily detectable through ctDNA, even at small tumour volumes. Given that liver is the initial metastatic site in almost all UM cases, ctDNA would be expected to detect metastatic disease in UM with high sensitivity.

Lastly in metastatic UM, studies utilising ultra-deep sequencing (without unique molecular identifiers (UMI) barcoding), detected ctDNA in approximately 41% and 80% of patients with metastatic UM [116, 121]. Other studies, used pyrophosphorolysis-activated polymerisation to detect ctDNA in 82–95% of patients with metastatic disease [89, 122–124], or ddPCR detecting ctDNA in 55–100% [81, 85, 125]. The level of ctDNA in metastatic UM has been shown to correlate with tumour volume [89, 122, 124], miliary hepatic metastases, quantity of CTCs, progression-free survival, and overall survival [89]. Furthermore, ctDNA has shown to be a superior marker of survival prediction compared to CTCs [89] in metastatic UM.

Another promising clinical use of ctDNA in metastatic UM is the monitoring of patient response during therapy [123, 125, 126]. Currently, the most comprehensive study monitoring ctDNA in UM patients throughout a therapeutic intervention showed that baseline ctDNA correlates with lactate dehydrogenase levels and the sum of the product of bi-dimensional diameters at baseline [125]. Furthermore, ctDNA rise preceded radiological progression of disease with a 4–10-week lead time [125]. Although there are currently no effective treatments for the long-term survival of UM, as future treatments become available ctDNA could be a vital clinical marker of treatment failure and enable the early switch of treatments. In addition, its inclusion in clinical trials could provide early evidence of response expediting drug development.

There are many important considerations when assessing ctDNA (Table 2). Firstly, increasing the plasma/serum or several mutational targets would increase the sensitivity of ctDNA detection [127]. In general, most studies in UM outside of those using next-generation sequencing (NGS) panels, such as Park et al. 2021 or Francis et al. 2021, assess only the known driver mutations using targeted approaches such as ddPCR or bi-PAP rather than also encompassing other recurrently mutated genes such as *BAP1, SF3B1* or *EIF1AX*. Another important pre-analytical consideration is the choice in blood tube. Firstly, plasma is generally regarded as the preferred medium as research has shown that serum contains larger DNA fragments, likely due to apoptosis of haematopoietic cells during clotting; unique ctDNA molecules and variant allele frequencies are lower in serum; and rarer mutant ctDNA copies are undetectable in serum when compared to plasma [128]. While plasma is generally opted for, there are many different tube types that affect the detection of ctDNA. Historically, EDTA has been the preferred solution [129], especially over heparin which is known to inhibit Taq polymerase. Newer developments have incorporated EDTA and some form of fixative to extend the window in which plasma is still viable for ctDNA analysis. Previous research has shown that apoptosis of leucocytes in EDTA tubes is apparent by 24 hours at room temperature and two days at 4 °C [130]. Whereas in fixative tubes, such as Streck cfDNA BCTs, even after seven days at room temperature there is no evidence of gDNA contamination from lysed leucocytes [130]. Lastly, double-centrifugation, choice of extraction kit, and elution volumes have a measurable impact on the detection of ctDNA. The gold standard for ctDNA has generally been regarded as the Qiagen Circulating Nucleic Acid Kit, which has been consistently shown to recover the most cell-free DNA [131, 132]. It is apparent that in UM studies have mostly opted for EDTA blood tubes and Qiagen extraction kits, however, elution volumes have been variable. There is some evidence to suggest that increasing elution volumes improves the overall DNA yield from Qiagen columns [133].

### Circulating Micro RNA

Another circulating nucleic acid that could serve as a blood biomarker is miRNA (Fig. 3a–c). MicroRNAs are a family of small non-coding RNA that can regulate gene expression post-transcriptionally. Compelling evidence has demonstrated that dysregulated miRNA expression is an intrinsic feature of human cancers. The first report of miRNA dysregulation in cancer came from B-cell lymphoma, where the authors found that deletions to 13q14 in B-cell lymphoma caused down-regulation of miRNAs-15 and 16 [134]. These two miRNAs were later found to affect cell proliferation [135]. Since then, many studies have demonstrated that the expression of miRNAs is dysregulated in different tumours [136]. Cancer cells with abnormal miRNA expression evolve the capability to sustain proliferative signalling, evade growth suppressors, resist cell death, activate invasion and metastasis, and induce angiogenesis [136]. Studies in many different cancer types and stages have shown that cmiRNA might be useful in either a diagnostic or prognostic capability in breast (miR-21), colorectal (miR-21), non-small cell lung (miR-21), or prostate (miR-375) cancer, and melanoma (miR-221) [137].

Worley *et al*. found upregulation of miRNA- let-7b, 143, 199a, 199a*, 193b, and 652 in primary UM tissue, which could discriminate patients with class 2 from class 1 with 100% sensitivity and specificity [138]. Recent advances in the genetic profiling of primary UM tumours revealed four distinct prognostic classifications [23]. Within these molecular subsets, four main miRNA expression clusters were found. These miRNA expression clusters were associated with monosomy 3 and DNA methylation [23]. As the levels of miRNA expression appear to correlate with prognostic classes within the primary tumour, plasma/serum levels of miRNA might also reveal prognostic information. Reports of cmiRNA found differentially expressed in UM patients described in Table 3. However, only one study, so far, has reported on the prognosis value of plasma miRNAs in primary UM, indicating that elevated plasma levels of miRNA-96b, miR-199a-5p and miR-233 were detected in patients with monosomy 3 [139].

**Table 3:** cmiRNA studies in UM.

Study	System	Study Type	Blood Tube	Blood Volume	TTP (hrs)	Centrifugation (xg/min)	Extraction Kit	Elution Vol (µL)	Patient numbers	miRNA found
Achberger [141]	RT-PCR	P	NS, plasma was used	NS, plasma was used	NS	NS	miRNeasy	NS	6 pUM	Increased at baseline compared to controls: miR-20a, 125b, 146a, 155, 181a, 223 Increased on mUM: miR-20a, 125b, 146a, 155, 223 Decreased on mUM: miR-181a
Ragusa [143]	Discovery: miRNA microarray Validation: RT-PCR	P	Dry vacutainer	400 µL serum	≤2	3,000 rpm/15 → −80 °C → 2000 rpm/10	miRNeasy	40	Discovery: 6 pUM Validation: 12 pUM	Serum miRNA differentially expressed: miR-30d, 127, 146a, 451, 518f, 523, 1274B
Russo [144]	miRNA microarray, RT-PCR	P	Dry vacutainer	400 µL serum	≤2	3000 rpm/15 → −80 °C → 2000 rpm/10	miRNeasy	40	14 pUM	Increased on pUM: miR-146a, miR-523 Decreased on pUM: miR-19a, 30d, 127, 451, 518f, 1274B
Triozzi [139]	miRNA microarray, RT-PCR	NS	NS	Plasma, volume not stated	NS	NS	miRNeasy	NS	20 pUM	Increased in patients with monosomy 3: miR-191, 93, 221, 342-3p, 19b, 199a-5p, 25, 27a, 23a, 15b, 223 Decreased in patients with monosomy 3: miR-1227, 663, 654-5p, 1238
Stark [140]	RT-PCR	P	SST	Serum, volume not stated	NS	1500/10	miRNeasy	NS	10 naevi, 50 pUM, 5 mUM	Serum miRNA differentially expressed between naevi, pUM & mUM: miR-16, 145, 146a, 204, 211, 363-3p Serum miRNA differentially expressed between pUM and mUM: miR-211

*TTP* time to process, Centrifugation in xg/min unless otherwise stated; *RT-PCR* reverse transcriptase-PCR, *P* prospective, *pUM* primary uveal melanoma, *mUM* metastatic uveal melanoma, *mIR* microRNA, *NS* not specified.

A study by Stark et al. [140], indicated that miR-211 levels were significantly elevated in serum of metastatic UM patients. In contrast, a previous study reported that plasma miR-146a, together with miR-20a, 125b, 155 and 223 were elevated when metastasis manifested [141]. Further studies are needed to validate these findings, as serum/plasma miRNA analysis may provide a simple strategy for monitoring recurrence in UM patients.

Although ophthalmologists can generally classify pigmented choroidal lesions into a naevi or a UM through various imaging modalities, some pathologies are difficult to differentiate and, hence, remain classified as indeterminate choroidal melanocytic lesions. In addition, the high prevalence of choroidal naevi (~5% of adults) poses a clinical burden, despite the low risk of transformation (<1%), with patients requiring yearly monitoring [142]. Multiple studies have described the potential of plasma/serum miRNA as a discriminator between healthy controls and patients with primary or metastatic disease. Differential expression of circulating miR-20a, 125b, 146a, 155, 181a, and 223 has been previously detected in patients with primary disease compared to healthy controls [141]. In addition, two independent studies have also identified upregulation of miRNA-146a in the serum of patients with UM relative to healthy controls [143, 144]. Recently, we reported that serum miR-146a, as well as miR-16, miR-145, miR-204, miR-211 and miR-363-3p, could differentiate patients with a benign uveal naevus from patient with primary UM with 93% sensitivity and 100% sensitivity [140]. However, the number of participants was low and requires further validation. In general, blood-based analysis of cmiRNA in UM is lacking and to date, the only miRNA consistently reported to increase with the development and progression of UM is miR-146a.

There are a few considerations for their analysis and interpretation of cmiRNA (Table 3), and currently one of the largest challenges in the field is their reproducibility. Similar to ctDNA, the choice of bio-fluid can impact the interpretation of results. Most cmiRNA studies have utilised serum, and serum may cause miRNA to be released from blood cells during the coagulation process [145]. Whereas platelet contamination may be an issue in plasma samples but can be removed via further centrifugation steps [146]. Therefore, it is important to keep in mind sample type when comparing across sample types. Again similarly to ctDNA as described above, the choice of extraction kit is important and again Qiagen miRNA mini kits appear to the most promising option currently for consistency and yield [147]. Many studies have utilised large scale miRNA microarrays for discovery, and previous research has shown that while intra-platform repeatability is high, inter-platform concordance is poor [148], and it appears that small RNA-seq is arguably the best overall method [149] but has not been utilised in UM to date. Lastly, data normalisation has been an area of debate whether to use the global mean expression or synthetic spike in controls such as *c. elegans* cel-miR-39, and results been methods should be carefully compared.

Studies assessing cmiRNA in UM currently lack method standardisation, even more so than those in CTCs and ctDNA. The results are difficult to compare and the reproducibility of individual cmiRNA seem unclear. Future studies validating current findings are needed to fully elucidate cmiRNA’s ability to provide diagnostic or prognostic information. Overall there is a lack of substantive evidence on the clinical validity of cmiRNA as a biomarker for UM. However, the existing reports suggest that some cmiRNAs may serve as an indicator of early malignant transformation of ocular naevi into primary UM.

### Exosomes

Both serum and plasma contain small extracellular vesicles known to contain DNA, RNA, miRNA and proteins [150]. To our knowledge, only three published papers and one published abstract investigate exosomes as a primary focus in more than 1–2 patients in UM from the blood. For example, in Ragusa et al. 2015 comparison of serum cmiRNA to vitreous humour and vitreous humour exosomes in primary UM and revealed that of 27 differentially expressed exosomes in the vitreous exosomes, 6 miRNAs matched both the vitreous humour and vitreous humour exosomes. Of these, miR-146a appeared to be the most elevated among the three sample types [143]. Eldh et al. isolated exosomes from liver perfusate of metastatic UM patients undergoing isolated hepatic perfusion [151]. The authors found upregulation of several miRNAs of which only miR-125b and 25 have been previously reported as differentially expressed in metastatic or poor prognosis UMs [139, 141]. Next, Frenkel et al. found that the mean size of exosomes were larger in those that developed metastases over those who were on observation [152]. Lastly, Wróblewska et al., 2021 using the Bio-Plex 37-plex human inflammation immunoassay assessed the protein profile of serum exosomes in primary and metastatic UM. The authors found that metastatic UM had significant enrichment of inflammatory markers, such as interferon γ, when compared to primary UM, and thus may potentially be suitable for detecting metastatic UM [153]. Other studies have combined uveal or ocular melanoma [154, 155], thus no strong conclusions about UM can be drawn. However, it does appear that potentially capturing UM exosomes via MCSP immunomagnetic beads may be more informative [155] rather than overall profiling. Currently, due to the lack of studies, there is little evidence of the clinical utility of exosomes in UM and future studies are needed to investigate them more thoroughly as markers.

## Future directions and conclusions

**Fig. 4:**
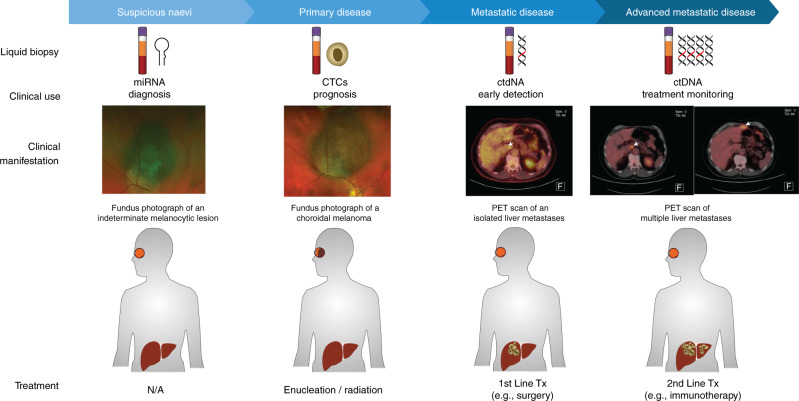
Suggested applications of liquid biopsy for the management of uveal melanoma. Plasma cmiRNA profiling could be used to distinguish a suspicious naevus from a small UM lesion. Upon diagnosis of UM, analysis of CTCs can provide information on prognostically relevant SCNAs. After treatment of the primary tumour, regular monitoring of plasma ctDNA could serve as an early indicator metastatic disease. Plasma ctDNA can be also used for monitoring of response to treatment and disease progression in patients with metastatic disease.

Over the last few decades, we have gained a better understanding of the biology of UM. However, significant improvements in the clinical outcome of this disease are still lagging. Like with all cancers, early detection and early treatment could greatly improve survival outcomes. In this regard, minimally invasive blood tests have a great potential to improve early diagnosis of primary UM, disease prognostication and detection of metastatic lesions, enabling timely and less radical therapeutic interventions (Fig. 4).

Based on our current knowledge, different types of liquid biopsies can serve different purposes. For example, serum miRNA may be useful for the identification and differentiation of benign melanocytic lesions from primary UM. This approach would help alleviate patient’s concerns after diagnosis, and more importantly, it would allow for earlier interventions, if UM is the confirmed condition. Treatment of small UM lesions has several advantages, such as allowing for sight preserving therapy and reducing the risk of metastases [5].

Following diagnosis of UM, CTCs could be used as a minimally invasive prognostic test to establish the risk of metastasis. Currently, the use of tumour biopsies for prognosis purposes varies significantly between practices around the world, generally affected by fears of poor sight altering outcomes. Using a minimally invasive prognostic test, such as CTCs, would improve clinical outcomes by allowing more efficient monitoring of high-risk UM patients, potentially enabling earlier surgical or therapeutic interventions. Furthermore, CTC testing could serve as a marker to select patients for adjuvant clinical trials. Currently, various targeted therapies and immunotherapies for UM are undergoing clinical trials (http://www.clinicaltrials.gov). While these trials herald a new and promising era with more effective treatments, it remains crucial to improve the early identification of high-risk patients.

Lastly, ctDNA testing could serve as regular monitoring modality for the early detection of metastatic disease. This test could be performed routinely, to function as a complementary test or as an alternative to expensive and invasive radiological scans. Earlier detection of metastases will enable more timely treatments or surgical interventions, both critical to improve patients’ outcomes.

CtDNA could also serve as a suitable method of minimally invasive treatment monitoring for metastatic UM and could be used to inform clinicians of changes to tumour burden and, potentially, early evaluation of response in future clinical trials. In particular, ctDNA sequencing may help to identify a proportion of patients likely to respond to immune checkpoint blockade. For example, although UM carries low number of mutations, iris melanomas have been shown to carry high tumour mutational burden which may be predictive of response to immunotherapies [43]. In addition, evidence suggests that patients with *MBD4* mutations respond to immune checkpoint blockade [126], likely due to the increased tumour mutational burden produced by the defect which causes wide-scale deamination of 5-methylcytosine (CpG to TpG).

In the future, liquid biopsies may be able to answer even more complex questions regarding UM progression. For example, recent studies have started to unravel the complexities of the tumour microenvironment, at a single-cell level, in primary and metastatic UM. This may one day lead to the discovery of new targets for therapy [156]. Such studies are possible thanks to novel single-cell RNA sequencing technologies, which could also help with the profiling of CTCs, to evaluate transcriptional changes that enable their dissemination throughout the body and the successful metastatic colonisation of the liver. On the other hand, ctDNA could be used to monitor genetic and epigenetic changes [157] during UM tumour development as they adapt and become resistant to treatment, as has been shown for other cancers. Altogether, the current evidence strongly suggests that liquid biopsies are a promising tool in UM to obtain detailed and valuable tumour information that can improve disease management through a personalised treatment selection, but these markers still require further investigation due to the lack of standardisation of methodologies and pre-analytical factors. Due to the rarity of the disease, large-scale multi-centre trials are needed to fully validate liquid biopsies in UM.

## Supplementary information

Supplementary Table 1
