# Supplementary material for: Future perspectives of uveal melanoma blood based biomarkers
Source: Br J Cancer. 2022 Feb 21;126(11):1511–28. doi: 10.1038/s41416-022-01723-8 (PMC9130512; doi:10.1038/s41416-022-01723-8)
Supplement: Supplementary file 1 — Supplementary Table 1 [file 41416_2022_1723_MOESM1_ESM.docx]

**Supplementary Table 1: Indirect Measurement (RT-PCR) CTC studies in UM**

| **Study** | **Sample)** | **Identification Marker** | **Blood Tube** | **Blood Vol (mL)** | **Time to Process (hrs)** | **Detection Rate** | **Summary of outcomes** |
| --- | --- | --- | --- | --- | --- | --- | --- |
| Tobal 1993 ^70^ | WB | *Tyrosinase* | NS | 5 | NS | pUM  1/4  mUM  2/2 | *Tyrosinase* mRNA was detected in both cases with metastases. The mRNA was also detected in one case that developed metastases 9 months later. |
| Foss 1995 ^71^ | WB | *Tyrosinase* | EDTA | 3.2 | ≤4 (held at 4°C) | pUM  0/36 | No positive results in 51 samples from 36 patients. There was no detection from perioperative samples either. |
| El-Shabrawi 1998 ^72^ | WB* | *Tyrosinase* | NS | 5* | NS | pUM  2/12 | Cites Tobal et a., 1993 for methodology. First positive case required enucleation; second positive case developed liver metastases 12 months later. |
| Keilholz 2004 ^73^ | WBCs | *Tyrosinase, gp100, MART1* | EDTA | 10 | ≤2 | pUM  3/24 *Tyrosinase*  1/24 gp100  1/24 *MART1*  mUM  24/40 *Tyrosinase*  4/40 gp100  32/40 *MART1* | Numbers refer to samples positive, some patients had multiple samples.  2/3 patients with positive PCR at primary developed liver metastases within the study period. 1/17 with a negative PCR developed liver metastases. |
| Boldin 2005 ^74^ | WBCs | *Tyrosinase* | H | 15 | ≤0.5 | pUM  16/41 baseline  5/16 post-treatment | Detection of CTCs at baseline correlates with 5-year survival. |
| Schuster 2007 ^75^ | WBCs | *Tyrosinase, MART1* | EDTA^ | 10 | ≤2 | pUM  5/110 *Tyrosinase*  5/110 *MART1*  1/110 *Tyrosinase + MART1* | Recruited those with clinical features of poor prognosis. RT-PCR positivity predictor of metastases (Hazard Ratio 7.3). |
| Callejo 2007 ^76^ | PBMCs | *Tyrosinase, MART1* | EDTA | 20 | ≤0.5 | Various stages  29/30 detectable at some point | 5/30 patients enrolled prior to treatment; 25 after average 3.5 years between treatment and enrolment. Blood was collected 3 monthly after enrolment. |
| Pinzani 2010 ^77^ | WB | *Tyrosinase* | PG | 2.5 | ≤4 | pUM  20/41 | *Tyrosinase* levels significantly correlated with tumour dimension, disease free and overall survival. |
| Schuster 2011 ^78^ | WBCs | *Tyrosinase, MART1* | EDTA^ | 10 | ≤2 | mUM  43/68 | PCR results and LDH levels were independent prognostic factors of PFS and OS. Combination of PCR and LDH stratified survival well. |
| Charitoudis 2016 ^79^ | WBCs | *Tyrosinase, MART1* | EDTA | 12 | ≤2^ | pUM  Pre-treatment  2/184 *Tyrosinase*  20/184 *MART1*  Post-treatment  4/180 *Tyrosinase*  25/180 *MART1* | Surgery did not increase the number of CTCs released into the blood. |
| Soltysova 2020 ^80^ | Rosette-sep depleted WBCs | *SNAI1, SNAI2, TWIST1, ZEB1, KRT19* | EDTA | 10 | ≤4 | pUM  0/34  mUM  0/5 | EMT associated genes were unsuccessful in detecting CTCs in UM. Levels were compared to healthy controls. |

NS – not specified; WB – Whole blood; WBCs – White Blood Cells; PBMCs – Peripheral Blood Mononuclear Cells; RT-PCR – Reverse Transcriptase PCR; pUM – primary uveal melanoma; mUM – metastatic uveal melanoma; H – heparinised; EDTA - Ethylenediaminetetraacetic acid; PG – PAXgene; PFS – progression free survival; OS – overall survival; LDH – Lactate dehydrogenase; Venous blood was used in all studies; All studies were prospective; * Assuming based on Tobal *et al*, 1993; ^ Assuming based on Keilholz *et al.,* 2004.
